# Ex Situ Conservation, DNA Barcoding and Enzymatic Potential Evaluation of Macrofungi (Basidiomycota, Ascomycota) from Vietnam

**DOI:** 10.3390/jof11010034

**Published:** 2025-01-04

**Authors:** Nadezhda V. Psurtseva, Anna A. Kiyashko, Svetlana V. Senik, Thi Ha Giang Pham

**Affiliations:** 1 Laboratory of Fungal Biochemistry, V. L. Komarov Botanical Institute of the Russian Academy of Sciences, Prof. Popov Str., 2, St. Petersburg 197022, Russia; anna.kiyashko@binran.ru (A.A.K.); senik@binran.ru (S.V.S.); 2 Joint Vietnam-Russia Tropical Science and Technology Research Center, Nguyen Van Huyen, Nghia Do, Cau Giay, Hanoi 122100, Vietnam; giangvietnga@gmail.com

**Keywords:** Agaricomycotina, strains, LE-BIN culture collection, protected areas, taxonomical analysis, ITS verification, enzymatic activities, oxidases, cellulases, proteinases

## Abstract

The diversity and resource potential of macroscopic fungi in tropical regions remain understudied. Vietnam, being in a biodiversity hotspot, has a large number of new fungal species that are of interest for biotechnology and medicine. The presence of a large number of protected areas in Vietnam creates favorable opportunities for the study and ex situ conservation of tropical biodiversity. From 2012 to 2023, 785 strains of macrofungi from National Parks of Vietnam were preserved in the LE-BIN collection, 327 of which were barcoded with the sequences deposited in the NCBI GenBank. A taxonomic analysis demonstrated that many of the preserved isolates are potentially new or poorly studied species, representing a useful resource for taxonomical studies and a search for new medicinal mushrooms. More than 180 strains were studied for the first time for growth rate and enzymatic activities. Of these, 53 strains showed high growth rate, 43—high cellulolytic activity, 73—high oxidative enzymes activity, and 27 showed high proteolytic activity, making them promising candidates for biotechnological and medical applications and opening new opportunities for sustainable biomass management, discovery of new enzymes and bioactive substances, development of new drugs and efficient plant waste treatment technologies. The results confirm the importance of the ex situ conservation of fungal diversity in tropical regions as a valuable source for scientific and commercial applications and suggest certain new active strains for biotechnological study.

## 1. Introduction

Tropical regions are among the most diverse and ecosystem-rich places on the planet, making them a source of unique biological resources. Fungi are an important part of these ecosystems, playing a key role in nutrient cycling, decomposition of organic matter, and symbiotic or parasitic interactions with plants. However, despite their importance, the diversity of tropical fungi and their potential biological activities remain poorly understood. Due to their genetic and ecological diversity, tropical fungi may possess a unique set of metabolites that could prove useful in biotechnology, medicine, and agriculture [1,2,3,4,5]. As estimated in recent studies, fungi, including macromycetes, have a significant impact on the global economy and their market presence will increase due to the rapid development of technology and related industries [6]. The significant financial value of mushrooms as well as their ecosystem importance indicate the need to conserve their habitats, i.e., the natural landscapes of various regions, including Southeast and Central Asia, for protecting undiscovered mushroom resources that will be lost if their habitats are destroyed [7]. Vietnam is located within the Indo-Burma Biodiversity Hotspot, which is one of the most biologically important regions of the planet in terms of species diversity and endemism, and is ranked in the top ten hotspots for irreplaceability and in the top five in terms of threat, with only 5% of its natural habitat remaining and more people living there than in any other hotspot [8,9]. Many new fungi have been described in the last 10 years, especially in tropical areas, for example, Hyde et al. [10] showed that up to 96% of fungi in northern Thailand, which is also located within the Indo-Burma Biodiversity Hotspot, may be new to science. The high diversity of plants and their communities in Vietnam suggests a high diversity of fungal species as well. The presence of a large number of protected areas: national parks (NP) and nature reserves (NR), creates favorable opportunities for the study and conservation of tropical biodiversity. However, the diversity of macrofungi in Vietnam is still not well studied and a large number of new mushroom species remain undescribed.

In the context of global warming and climate change, the study of fungi from tropical regions is particularly important. The increases in temperature and precipitation predicted for temperate regions in the coming decades could significantly affect ecosystem processes and biodiversity [11], making the study of fungi adapted to hot, humid climates essential. These organisms may have unique traits that allow them to survive and adapt to new climatic realities, which could be crucial for ecosystem conservation and resource use [12].

Ex situ conservation (conservation off-site) is one of the effective ways for preservation fungal resources. In addition, it creates the opportunity to study mushrooms in laboratory [13]. Long-term conservation of pure cultures carried out in culture collections (CCs), genetic resource collections or biological resource centers is most reliable and productive [14,15].

Komarov Botanical Institute Basidiomycetes Culture Collection (LE-BIN) has been preserving macromycetes ex situ since the late 1950s. Currently, it is the largest culture collection in Russia maintaining the taxonomic and ecological diversity of basidial and marsupial macrofungi [16]. Focused work in the LE-BIN culture collection on ex situ conservation of tropical macromycetes started in 2012 during an expedition to Cat Tien, Bidoup—Nui Ba and Phu Quoc National Parks in Vietnam. Since then, collection trips to various national parks and nature reserves in Vietnam have been regularly conducted to collect and cultivate macromycetes. Growth and morphological characteristics as well as some biochemical properties of several LE-BIN strains from Vietnam have been published. For example, the cultural and morphological characteristics of strain LE-BIN 2379 of the luminescent fungus were obtained. The amplified ITS nrDNA fragments were cloned, sequenced, and analyzed phylogenetically. The resulting culture was identified as *Neonothopanus nambi* (Speg.) R.H. Petersen and Krisai, a luminescent wood-destroying fungus first collected in South Vietnam [17]. Culture characters in ontogenesis of an interesting tropical basidiomycete fungus, *Rogersiomyces malaysianus* (K. Matsush. and Matsush.) Zmitr. were studied for the first time using strain LE-BIN 3507. In laboratory experiments, this strain demonstrated the ability to produce acetylenic acids. It contained triglycerides of 40% esterified with acetylene dehydrocrepenynic fatty acid. The culture demonstrated active growth and accumulated up to 25% of triglycerides from the dry mass of the mycelium (without technological optimization) [16,18]. The thermoplasticity of two strains of the tropical luminescent species *Favolaschia manipularis* (Berk.) Teng (*≡Filoboletus manipularis* (Berk.) Singer) LE-BIN 3272 and LE-BIN 3291 were studied to reveal the involvement of membrane lipids in the fungus adaptation to temperature changes [19]. However, until now, we have not summarized information on Vietnamese strains maintained ex situ in the LE-BIN collection.

The aim of this paper is to analyze the diversity of the ex situ preserved tropical fungi and assess their growth in culture and biosynthetic potential.

## 2. Materials and Methods

### 2.1. Origination and Isolation of Strains

**Figure 1:**
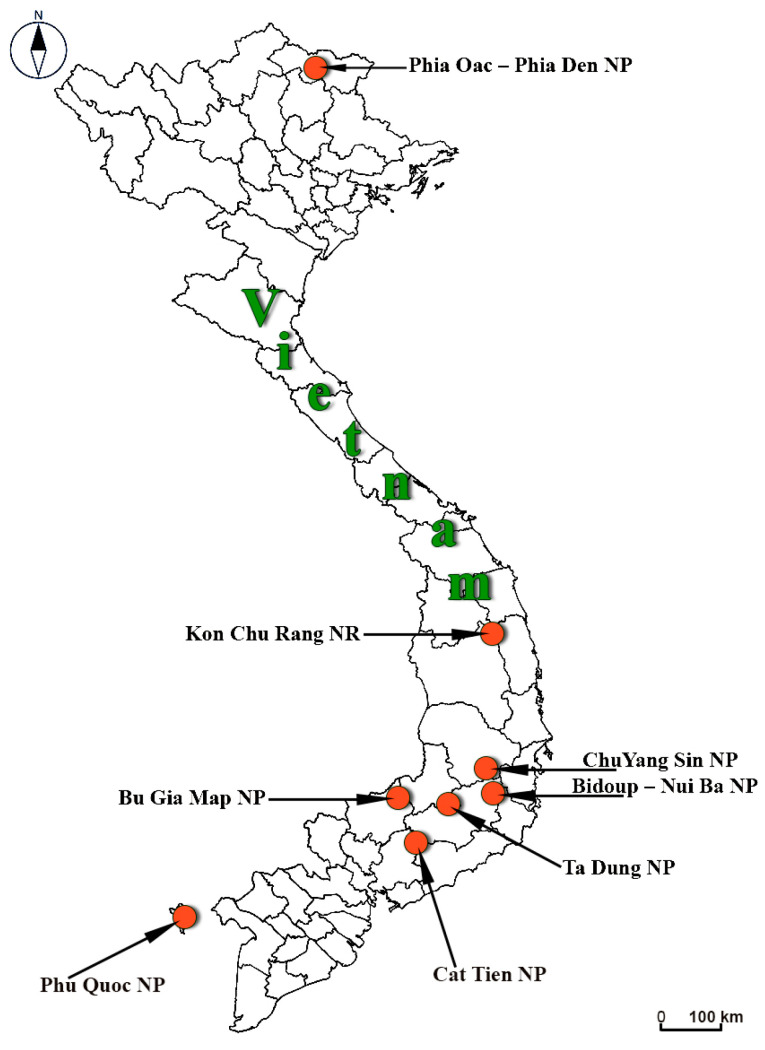
Protected territories in Vietnam where the ex situ conservation of macromycetes has been carried out.

The maintaining strains are original isolates from basiomata collected in NR Kon Chu Rang and NP of Vietnam from 2012 to 2023 (Table 1), situated in Northern (Phia Oac—Phia Den), Central (Cat Tien, Bidoup—Nui Ba, Kon Chu Rang, Bu Gia Map, Chu Yang Sin, Ta Dung) and Sothern (Phu Quoc) parts of Vietnam (Figure 1).

Fungal isolates were obtained manually from fresh basidiomata using their tissues or spores. Sterile plastic Petri dishes (Ø35–40 mm) filled with sterilized (121 °C, 15 min) beer-wort or malt extract (4%), agar (2%), supplemented with an aqueous solution of antibiotic (0.06% kanamycin or gentamicin) were used. When the mycelium growth or spore germination started, a small piece of growing culture (about 2 mm^2^) was transplanted into 2 mL sterile plastic microvials with 0.7 mL of the medium for further growth and transportation.

### 2.2. Preservation of Strains

The preservation of strains in the LE-BIN collection was carried out by three methods simultaneously, as described before [16]: subculturing in tubes on beer-wort agar slants in a refrigerator 14–16 °C; disk method in screw-cap microvials under distilled water at 14–16 °C; cryopreservation method in screw-cap cryovials in a 10% aqueous glycerol solution at −80 °C, with a freezing rate of 1° C min^−1^.

### 2.3. Strains Verification

#### 2.3.1. Cultivation and Morphological Methods

The LE-BIN tropical strains were cultivated on beer-wort agar (BWA)—4% original beer-wort from breweries (St. Petersburg, Russia) and agar 20 g/L (Difco, Sparks, MD, USA) and malt extract agar (MEA)—15 g/L malt extract (Conda, Madrid, Spain) and agar 20 g/L (Difco, Sparks, MD, USA). Sterilization was performed for 30 min at 121 °C.

The morphology of colonies was described using traditional diagnostic characters and terminology [20]. Macromorphology: (a) the advancing zone and the outline of the colony; (b) the texture of the mycelial mat; (c) the colony color; (d) the odor; and (e) the reversum (reverse side) of the colony. Micromorphology: (a) presence of clamps; (b) width and (c) type of hyphae; (d) presence of various hyphal structures, like anastomoses, hyphal rings, hyphal strands, rhizomorphs and sclerotia, cystidia and gloeocystidia, encrusted hyphae, crystals, hyphal nodes, various swellings, etc.; and (e) the presence of propagative structures: arthroconidia, conidia, chlamydospores, blastoconidia, etc. Sometimes, these structures have taxonomic significance [20,21]. The colony morphology was examined after 2 and 4 weeks. Micromorphology was studied under Zeiss Axio Imager A1 and Axio Scope A1 (Zeiss, Oberkochen, Germany) using transmitted light.

#### 2.3.2. Molecular Methods

Verification of the strains was performed by PCR analysis of the nuclear ITS (and, occasionally, LSU) rDNA region (fungal barcode), sampling a small piece of mycelium from the advancing zone of the colonies and using Thermo Scientific Phire Plant Direct PCR Kit (Vilnius, Lithuania) and standard basidiomycetes primers ITS1F and ITS4B (or ITS4) for amplification. Primer ITS4 was applied mostly for Auriculariales (Agaricomycetes), Tremellomycetes, Dacrymycetes, and marsupial fungi. Primers LR0R and LR5 were used for LSU (https://www2.clarku.edu/faculty/dhibbett/protocols_folder/primers/primers.pdf, accessed on 23 September 2024). In some cases, DNA was extracted using the Nucleo Spin Plant II (Macherey-Nagel, Duren, Germany) or similar kits. Sequencing was performed with an ABI model 3130 Genetic Analyzer (Applied Biosystems, Foster City, CA, USA) using the BigDyeTM Terminator Cycle Sequencing Ready Reaction Kit (AB) at the Core Facility Centre of the Komarov Botanical Institute RAS. Raw data and the final sequences were processed using MEGA 7—MEGA XI [22,23]. A search for closely related sequences was performed using the BLAST program in the NCBI public database (https://www.ncbi.nlm.nih.gov, accessed on 4 December 2023–4 November 2024). Generated sequences were deposited to the GenBank NCBI.

### 2.4. Taxonomical Analysis

The nomenclature and systematic position of the collection’s strains is given according to the Index Fungorum and MycoBank databases as well as He et al. [24,25], Wijatawardene et al. [26], and Kalichman et al. [27], taking into account a number of modern publications concerning some taxonomic groups, e.g., [28,29,30,31,32,33,34,35,36,37,38].

Voucher specimens of strains were studied and preserved according to traditional methods used in mycology [39]. Macromorphological observations were based on fresh basidiocarps; their collecting was accompanied by taking in situ digital photos. Microscopic observations were made, when it was necessary, from dried material mounted in 5% KOH, 10% Congo Red in NH_4_OH, or Melzer’s reagents using a Zeiss Axio Scope A1 and Axio Imager A1 light microscopes with differential interface contrast (DIC). All examined specimens are deposited in the Mycological Herbarium of the Komarov Botanical Institute (Saint Petersburg, Russia).

### 2.5. Methods for Evaluation of Growth and Enzymatic Activity

Inoculum plugs (7 mm in diameter) were placed mycelium-side down on the edge of 60 mm Petri dishes containing BWA or MEA and incubated for 6 weeks in growth chambers in the dark at 25 °C to evaluate the growth rate of the strains. The experiments were conducted in three replicates. The growth rate was recorded every other day (or every day in case of fast-growing species), and linear mycelial extension (mm) and standard deviation were estimated using the MS Excel 2016 statistics tool. A growth rate of less than 4 mm/day was considered slow growth, 4 to 6 mm/day was considered medium growth, and more than 6 mm/day was considered fast growth.

To qualitatively evaluate the activity of oxidoreductases (ligninolytic enszymes) by the express method, strains inoculated as described above were grown in the dark at 25 °C in Petri dishes on BWA medium. Oxidase activity was determined after two weeks of growth. Mycelial plugs (3 replicates on each substrate) with a diameter of 7 mm were cut at the edge of the growing colony and transferred to the wells of microbiological plates, followed by a dropwise addition of guaiacol (GU, 2 mL/100 mL H_2_O) (Sigma, St. Louis, MO, USA) and 1.0% syringaldazine (SG, Sigma, St. Louis, MO, USA) in C_2_H_5_OH. The activity was evaluated visually by color reaction intensity on a scale from “−” (no activity) to “+++” (high activity) at intervals of 5, 30, and 60 min, and 3 h.

Proteolytic activity was evaluated using gelatin as a substrate (gelatinase activity, GA) consisting of sterilized distilled water and 4% food-grade gelatin heated in a microwave oven until dissolved, cooled to 40 °C, and poured into 90 mm-diameter Petri dishes. After the gelatin has hardened, three mycelium plugs (7 mm-diameter) were applied to its surface in one plate. The plates were incubated at 25 °C for 48 h; afterward, the zones of gelatin lysis were measured in two mutually perpendicular directions (mm). In case of high activity, the time of incubation was reduced up to 24 h to prevent zones fusion.

Cellulolytic activity (CA) was estimated using distilled water substrate consisting of 10.0 (g/L) microcrystalline cellulose (MCC; Chemapol, Praha, Czech Republic or Vecton, St. Petersburg, Russia) and 20.0 (g/L) agar (Difco, Sparks, MD, USA). Application and incubation were carried out as described above (25 °C for 48 h). The activity of cellulases was determined by the measurement in two mutually perpendicular directions (mm) of an enlightened zone around the inoculum. For zone development, the surface of plates was short-term treated with solution of 0.5% I in 2% of KI.

Each activity was assessed in three replicates. The average diameter of zones (mm) and standard deviation (*n* = 6) were estimated using the MS Excel 2016 statistics tool.

## 3. Results and Discussion

### 3.1. Ex Situ Conservation of Tropical Macromycetes

Since the work on ex situ conservation of tropical macromycetes was started in Vietnam in 2012, eight national parks were surveyed in different years to study and isolate fungi in pure culture (Table 1). In total, about 1500 macromycete specimens have been collected for culturing during the field trips over the years, and about 1000 isolates have been obtained and brought to the laboratory for further investigation. The results of this work for each national park are summarized in Table 2.

The difference between the number of samples collected and isolates obtained was due to the fact that many basidiomata did not drop spores in Petri dishes, or due to contamination by fast-growing molds. The number of successfully obtained strains decreased due to the lack of spore germination, growth absence, or contamination. As a result, the number of strains obtained is approximately half of the number of samples collected. A detailed discussion of the problem of obtaining and maintaining pure cultures of macromycetes in the field, as well as the ways of increasing the percentage of successfully obtained isolates, was presented in our previous work [16].

**Figure 2:**
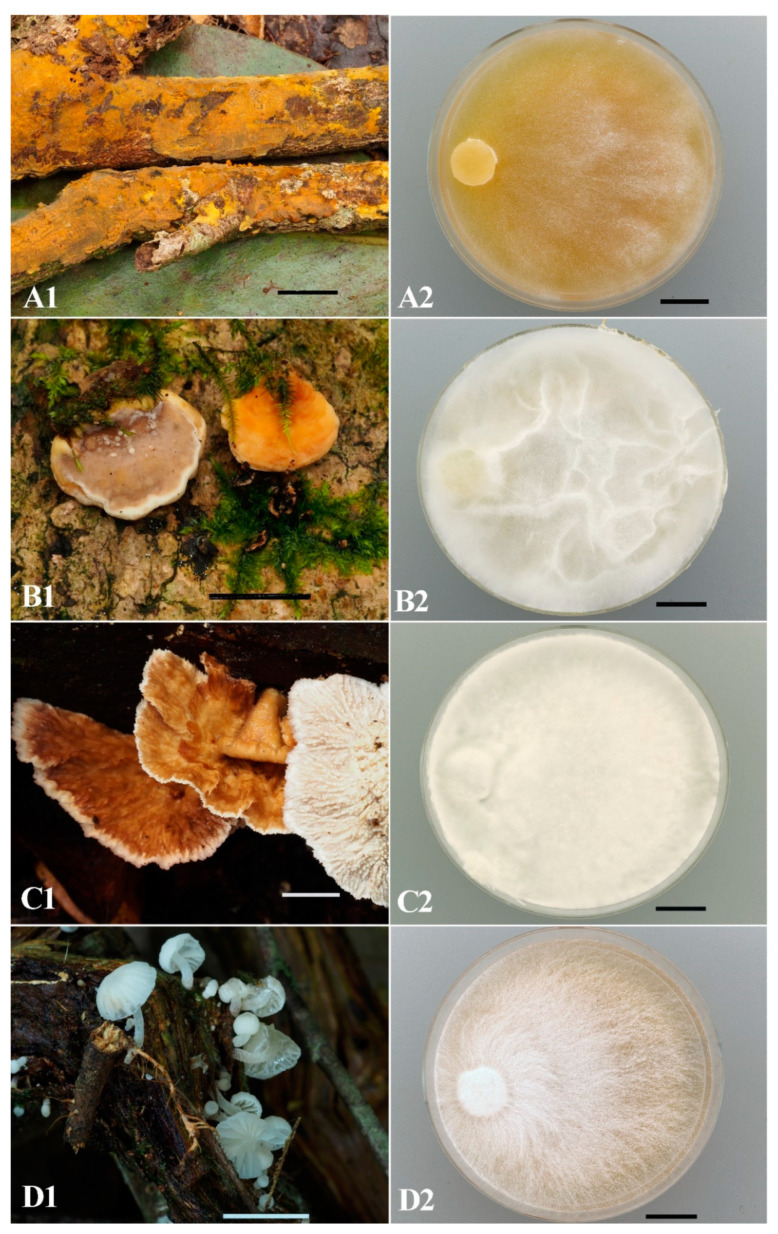
Natural basidiomata (voucher specimens) (**1**) and their strains on MEA (**2**): (**A**) *Crustodontia chrysocreas* LE-BIN 5340. (**B**) *Physisporinus lineatus* LE-BIN 5342. (**C**)—*Cymatoderma elegans* LE-BIN 5356. (**D**) *Mycena jingyinga* LE-BIN 5377. Scale bars are 10 mm.

**Figure 3:**
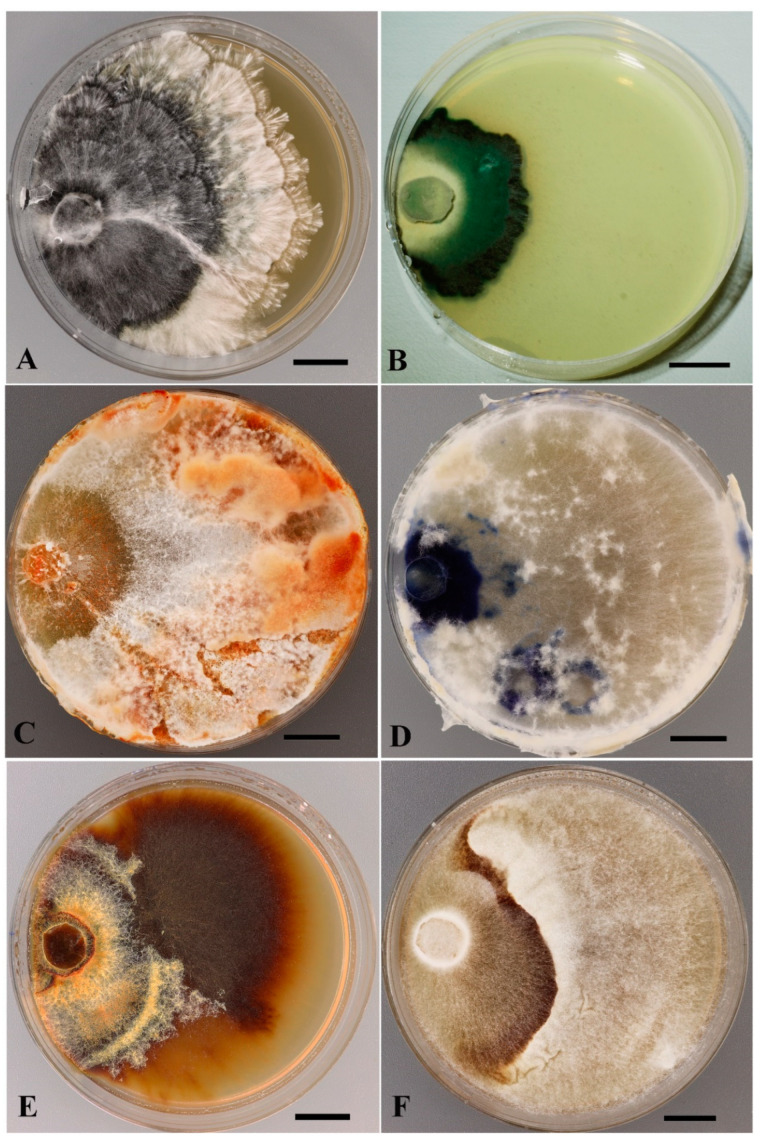
Colony mats (3 weeks) with specific macromorphological characters. (**A**) *Xylaria grammica* LE-BIN 5102. (**B**) *Chlorociboria* sp. LE-BIN 4294. (**C**) *Trametes sanguinea* LE-BIN 5053. (**D**) *Terana* sp. LE-BIN 5152. (**E**) *Hymenochaete rheicolor* LE-BIN 5115. (**F**) *Lentinus squarosulus* LE-BIN 5189. Scale bars are 10 mm.

However, the detailed study in the laboratory of the obtained isolates revealed a number of non-germinated spore prints, as well as non-growing and contaminated isolates. Based on cultural studies, the isolates were verified and introduced into the LE-BIN collection. The number of strains from each national park is presented in Table 2. As can be seen in the table, quite a large number of isolates were rejected as a result of cultural studies. Examples of natural specimens of macromycetes collected in 2023 from Bidoup Nui Ba (A-C) and Kon Chu Rang (D) national parks and their mycelial colonies are shown in Figure 2. Colonies of some species have specific colony mats that can be very useful in the verification of strains, to at least genus level. Several examples of such 3-week-old colonies are presented in Figure 3.

It should be noted that strains of some tropical mushroom species cannot withstand prolonged storage at 5 °C, which is commonly used for the preservation of mushrooms from the boreal zone. For example, it was found that the minimum temperature at which strains from Vietnam *Neonothopanus nambi*, *Favolaschia manipularis*, and *Rogersiomyces malaysianus* showed signs of growth was 10 °C. Nevertheless, the cryopreservation of such strains at −80 °C has been quite successful. For this reason, in the LE-BIN collection, strains from Vietnam are stored in subculture tubes and distilled water vials at 14–16 °C, while cryopreservation is performed at the standard temperature of −80 °C.

### 3.2. DNA Barcoding of LE-BIN Strains from Vietnam

DNA barcoding is an important component in the ex situ conservation of macromycetes cultures. This method allows the detection of strains contaminated with other basidiomycete fungi, as well as the clarification of the taxonomic affiliation of strains. In 2012, the International Fungal Barcoding Consortium formally recommended the use of internal transcribed spacers (ITS) of the nuclear ribosomal RNA gene as the primary fungal barcode. In fungi, the ITS region is about 600 bp long and contains two variable spacers, ITS-1 and ITS-2, which are separated by the highly conserved 5.8S rRNA gene [40,41,42,43]. In recent years, the use of a molecular approach based on DNA barcoding has become more and more demanded for the identification of fungi [44]. Culture collections and biological resource centers barcode preserved biological material as part of special funded projects. An example is the CBS fungal biobank in the Netherlands, one of the world’s largest centers for preserving fungal diversity in culture. At this center, the sequences of two nuclear ribosomal genetic markers, ITS and 26S Large Subunit (LSU), were obtained as DNA barcode data for about 100,000 fungal strains of about 17,000 species [45]. Despite the fact that the examination of barcoding results has revealed cases of ambiguous identification of fungi, and numerous mislabeled reference sequences have already been deposited in the NCBI gene bank, at the molecular level ITS remains a versatile barcoding marker for the primary identification of fungi or confirmation of species name. It was recommended that if ITS is insufficient for species discrimination, LSU, TEF1α or multilocus approaches should be used to achieve the desired level of accuracy and precision [40,46].

DNA barcoding of LE-BIN strains from Vietnam allowed revealing of strains contaminated with other macromycetes that were not detected at the stage of cultural study. Due to the molecular method, the contaminated strains were eliminated from the collection, and 327 strains were validated or identified. The generated sequences of ITS and LSU regions were deposited in the GenBank NCBI (https://www.ncbi.nlm.nih.gov/nuccore, accessed on 9 December 2022–4 November 2024). The deposited strains with the NCBI accession numbers are presented in the Supplementary Materials (Table S1). DNA barcoding of LE-BIN strains revealed a large number of unidentified and probably novel tropical fungal species that could not be identified by either the traditional morphological method or the megablast algorithm at NCBI due to the lack of highly similar sequences with more than 98% homology with our queries in GenBank. We identified such strains to genus and deposited their sequences using only their genus rank. For example, strain LE-BIN 5043 (GenBank Acc. Num. PQ555048) showed high homology in LSU region (99.07%) with recently described *Neohypochnicium yunnanense* J.H. Dong and C.L. Zhao (GenBank Acc. Num. OQ789003); however, ITS region (GenBank Acc. Num. PQ558619) demonstrated low similarity with this species: 92.80% and 94.52% with sequences OQ789011 and OQ789010, respectively. One of the other strains of taxonomic interest is *Terana* sp. LE-BIN 5152 (GenBank Acc. Num. OR885626; Figure 3D). To the best of our knowledge, according to modern nomenclature, this genus is monotypic, including one species of *Terana coerulea*, but the ITS-sequence of this strain is only 95.5–96.7% similar to *T. coerulea* in GenBank. Another interesting unidentified species is *Stereum* sp. collected in Bidoup—Nui Ba NP, Bu Gia Map NP, and Kon Chu Rang NR in different years and locations. Its strains LE-BIN 3043, LE-BIN 4203, LE-BIN 5359, LE-BIN 5379, and LE-BIN 5394 have high ITS sequence similarity indicating that they belong to the same species, but their homology with other GenBank sequences is 95% or less. The same situation was observed with strains of *Mycetinis* sp. LE-BIN 2995, LE-BIN 3053, LE-BIN 4589, LE-BIN 5374, and LE-BIN 5384 from Phu Quoc NP, Bidoup—Nui Ba NP and Kon Chu Rang NR: high similarity of ITS sequences between strains, but no homology with other GenBank sequences sufficient to identify these strains. We were also faced with the fact that some species within species complexes were problematic for identification using a molecular approach, e.g., *Trametes sanguinea*/*coccinea*, as was discussed in Lücking with coauthors [46], *Microporus* (≡*Trametes*) *vernicipes*/*xanthopus*, etc. Among the agaricoid fungi, strains of *Gymnopus*, *Collybiopsis*, *Mycetinis*, and *Mycena* can be distinguished that are difficult to identify by barcoding. The abundance of misidentified sequences in the GenBank is exacerbated by the presence of collection strains of potentially new species from these genera. Due to the fact that voucher specimens of basidiomata, from which cultures were isolated, are obligatorily stored in the mycological herbarium LE of the Komarov Botanical Institute, such strains together with their natural specimens can be used for further taxonomic studies.

### 3.3. Taxonomical Structure of the Vietnamese Fungi in the LE-BIN Collection

Currently, 801 strains (785 from the protected area) of Vietnamese basidial and marsupial fungi are stored in the culture collection of the Komarov Botanical Institute, of which 381 (47.5%) strains have been identified to species level and 312 (39%) to genus level. However, the taxonomic affiliation of 108 (13.5%) strains could not be established. Probably, they belong to yet undescribed species and genera.

All identified strains refer to more than 252 species and 179 genera of the two major subphyla encompassing macrofungi, i.e., Agaricomycotina (Basidiomycota) and Pezizomycotina (Ascomycota) (Table 3). However, Agaricomycotina makes the overwhelming majority of strains and species (appr. 95%), which is explained by the traditional direction of works in the collection [15]. Despite the small strain number, Pezizomycotina is presented by members of four classes, five orders, and nine families, from which the largest are Sordariomycetes (18 strains from more than 10 species) and Leotiomycetes (10 strains from more than 6 species). Among the Sordariomycetes, the most numerous in our collection are the family Xylariaceae (16 strains over 8 species) and the small but very important family Cordycipitaceae with two strains of two species, LE-BIN 5363 *Cordyceps ninchukispora* (C.H. Su and H.H. Wang) G.H. Sung, J.M. Sung, Hywel-Jones and Spatafora and *Isaria* sp.

Agaricomycotina is represented in the collection by three classes with the absolute majority of strains belonging to Agaricomycetes (657 strains out of 693 identified). The other two classes (Dacrymycetes and Tremellomycetes) include one–two strains each from the same genus (LE-BIN 4551 and LE-BIN 5051 *Dacrymymyces spathularia* (Schwein.) Alvarenga and LE-BIN 3319 *Phaeotremella foliacea* (Pers.) Wedin, J.C. Zamora and Millanes).

Currently, the Vietnamese strain collection represents all major phylogenetic lineages of Agaricomycetes (with the exception of Boletales) and encompass 10 orders, 58 families, and 163 genera. The two enormous orders Agaricales and Polyporales include about 80% of the identified strains. This arrangement is not surprising, as these orders are not only the largest in Agaricomycetes, but are formed predominantly by saprotrophic species that grow well in culture. The third major order Russulales is represented in the collection by 44 strains of also saprotrophic, mainly wood-destroying fungi. It is important to emphasize that the taxonomic composition and systematic structure of the Vietnamese strain collection do not so much reflect any biogeographic patterns but are related to the ease of obtaining isolates of different species and maintaining them in culture. Thus, the largest family Polyporaceae (135 strains more than 48 species) is composed of wood-destroying species, among them many common wide-distributed in tropics species: *Microporus xanthopus* (Fr.) Kuntze, *M. affinis* (Blume et T. Nees) Kuntze, *Trametes coccinea* (Fr.) Hai J. Li et S. H. He, *T. vernicipes* (Berk.) Zmitr., Wasser et Ezhov, *Cellulariella acuta* (Berk.) Zmitr. et Malysheva etc. Many of them form basidiocarps during the year including the dry season, actively sporulate, and can be easily isolated in culture. The second largest family, Omphalotaceae (68 strains of more than 15 species from 9 genera), is itself large, also formed by saprotrophic fungal species and includes the large genus *Collybiopsis*, which has the highest species diversity in tropical regions and is also easily isolated in culture. The same can be said about the Mycenaceae family (67 strains of more than 15 species from 7 genera), which also includes saprotrophic species widespread in tropical forests, as well as the very large genus *Mycena*, whose representatives can be isolated in culture rather easily. Genera represented by the greatest number of strains are also either large themselves (*Collybiopsis*, *Mycena*, *Marasmius*), or smaller but contain some very common species that are widely-distributed in Vietnam (*Microporus*, *Trametes*, *Pleurotus*, *Hypholoma*, *Gymnopus* etc.), or include biotechnologically important species (*Lentinus*, *Favolashia*, *Neonothopanus*, *Auricularia*) (Table 4).

It should be noted that we use the generic name *Marasmiellus* because for two species in the collection, *Marasmiellus rhizomorphogenus* Antonín, Ryoo et H. D. Shin and *M. scandens* (Massee) Dennis et D. A. Reid have not yet been made new combinations at the time of writing. In addition, in contrast to Corner [47], we preferred to retain the name *Vanromburghia silvestris* Holterm. versus *Trogia silvestris* (Holterm.) Corner, because the obtained sequence of ITS1-5.8S-ITS2 region of strain LE-BIN 5049 (OR683760) differs quite significantly from the sequences of typical representatives of the genus *Trogia*.

All of the most represented species are common in Vietnam and are widespread throughout the territory and in different forest types. The exception is *Neonothopanus nambi* (Speg.) R.H. Petersen and Krisai, which was actively collected due to our interest in studying its luminous properties.

Thus, the diverse taxonomic composition of Vietnam’s fungal culture collection can be valuable for various studies in systematics, biochemistry, and physiology of fungi, and can be applied to biotechnological developments.

### 3.4. Growth and Enzymatic Potential of LE-BIN Strains from Vietnam

A large set of Vietnamese strains isolated in 2018, 2019, and 2022 in Bu Gia Map, Phia Oac—Phia Den, Bidoup—Nui Ba and Ta Dung National Parks and Kon Chu Rang Nature Reserve were examined by express methods for linear growth rate and activity of lignocellulolytic enzymes. This is a large group of mainly extracellular proteins including ligninolytic enzymes (peroxidases and oxidases) and hydrolytic enzymes (cellulases, hemicellulases, pectinases, chitinases, amylases, proteases, esterases, and mannases). The activity of three groups of these enzymes—oxidative, proteolytic and cellulolytic, was the subject of our study. The results obtained for the strains studied are presented in the Supplementary Materials (Table S2).

**Figure 4:**
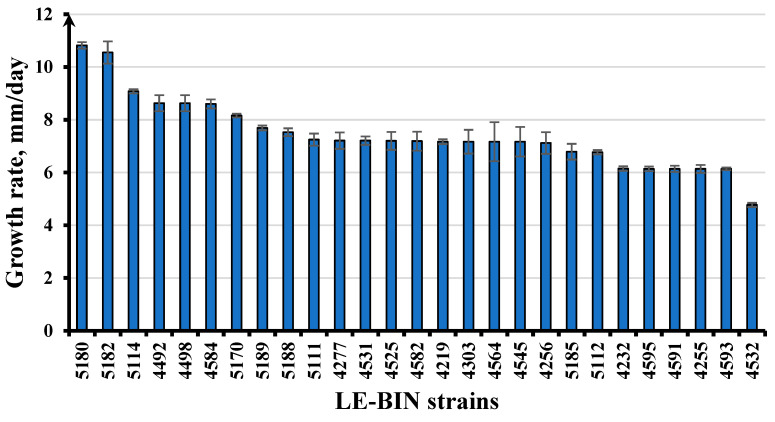
Growth rate (mm/day) of some fast-growing strains from Vietnam: *Tinctoporellus epimiltinus* LE-BIN 5180, *Trametes meyenii* LE-BIN 5182, *Oxychaete cervinogilva* LE-BIN 5114, *Cerrena zonata* LE-BIN 4492, *Phlebia acerina* LE-BIN 4498 and LE-BIN 4584, *Theleporus membranaceus* LE-BIN 5170, *Lentinus squarrosulus* LE-BIN 5189, *Pleurotus ostreatus* LE-BIN 5188, *Phlebiopsis crassa* LE-BIN 5111, *Bjerkandera adusta* LE-BIN 4277, *Fulvifomes fastuosus* LE-BIN 4531, *Trametes maxima* LE-BIN 4525, *Ganoderma applanatum* LE-BIN 4582, *Grammothele lineata* LE-BIN 4219, *Irpicodon* sp. LE-BIN 4303, *Psathyrella* sp. LE-BIN 4564, *Cellulariella acuta* LE-BIN 4545, *Trametes versicolor* LE-BIN 4256, *Hymenopellis* sp. LE-BIN 5185, *Candolleomyces* aff. *candolleanus* LE-BIN 5112, *C*. *acuta* LE-BIN 4232, *Trametes sanguinea* LE-BIN 4595, *Pleurotus pulmonarius* LE-BIN 4591, *Lentinus crinitus* LE-BIN 4255, *Conchomyces* sp. LE-BIN 4593, and *C*. *acuta* LE-BIN 4532.

The growth rate of a strain on agar medium is an important characteristic of fungal physiology, especially in terms of the use of macromycete strains in biotechnology. The Vietnamese strains studied differed in their growth rate, which usually correlates with their taxonomy—some species grow fast and can produce a lot of biomass in a short period, while others grow slowly and take much longer to cultivate. The growth of macromycete cultures depends on several factors, including temperature, culture medium, physiological state of the strain, etc., which means that it is possible to control the growth rate of the culture [16]. For example, it has been observed that after several repeated cultivations on a new Petri dish, the strain starts to grow faster. The growth experiments on Vietnamese strains were conducted in equal conditions without technological optimization because preliminary long-term experiments have shown that the optimal conditions for growth of the majority of basidiomycete strains are 25 ± 2 °C and native beer-wort or malt extract as a nutrient medium. In addition to the well-known and actively cultivated worldwide species *Trametes versicolor* (LE-BIN 4256), *T. maxima* (LE-BIN 4525), *T*. *sanguinea* (LE-BIN 4595), *Bjerkandera adusta* (LE-BIN 4277), *Ganoderma applanatum* (LE-BIN 4582), *Pleurotus ostreatus* (LE-BIN 5188), *P. pulmonarius* (LE-BIN 4591), *Candolleomyces aff. candolleanus* (LE-BIN 5112), whose rapid growth in culture was confirmed in our experiments (6.14–7.53 mm/day), strains of less cultivated species, including strains of recently described taxa, were characterized by fast growth (Figure 4). Among the aphyllophoroid fungi, *Trametes meyenii* LE-BIN 5182 (10.55 ± 0.42 mm/day), *Oxychaete cervinogilva* LE-BIN 5114 (9.08 ± 0.08 mm/day), *Phlebiopsis crassa* LE-BIN 5111 (7.25 ± 0.23 mm/day), *Theleporus membranaceus* LE-BIN 5170 (8.16 ± 0.07 mm/day), *Tinctoporellus epimiltinus* LE-BIN 5180 (10.82 ± 0.12 mm/day), *Grammothele lineata* LE-BIN 4219 (7.17 ± 0.09 mm/day), *Fulvifomes fastuosus* LE-BIN 4531 (7.21 ± 0.16 mm/day), *Cerrena zonata* LE-BIN 4492 (8.63 ± 0.30 mm/day), *Phlebia acerina* LE-BIN 4498 (8.63 ± 0.30 mm/day) and LE-BIN 4584 (8.6 ± 0.17 mm/day) and *Irpicodon* sp. LE-BIN 4303 (7.17 ± 0.45 mm/day) were noteworthy. Among the agaricoid fungi, *Lentinus squarrosulus* LE-BIN 5189 (7.69 ± 0.09 mm/day), *L. crinitus* LE-BIN 4255 (6.14 ± 0.15 mm/day), *Psathyrella* sp. LE-BIN 4564 (7.17 ± 0.74 mm/day), *Hymenopellis* sp. LE-BIN 5185 (6.79 ± 0.3 mm/day) and *Conchomyces* sp. LE-BIN 4593 (6.14 ± 0.05 mm/day) showed the most active linear growth under the studied conditions. *Cellulariella acuta* strains showed intraspecific diversity in growth rate, LE-BIN 4545 (7.17 ± 0.56 mm/day), LE-BIN 4232 (6.15 ± 0.09 mm/day) and LE-BIN 4532 (4.77 ± 0,08 mm/day). In general, strains of aphyllophoroid fungi showed a higher growth rate compared to agaricoid fungi. As expected, among *Panellus*, *Crepidotus*, *Collybiopsis*, *Mycetinis*, *Hypholoma*, *Gymnopus*, most of *Hymenopellis*, *Campanella*, *Termitomycea*, *Favolaschia*, *Pholiota*, and some other genera of agaricoid fungi, fast-growing strains were not found—all studied cultures showed slow or medium growth rates. Strains from some genera of aphyllophoroid fungi also grew slowly, such as *Microporus affinis* LE-BIN 4485 (1.87 ± 0.06 mm/day), *Vararia* sp. LE-BIN 4559 (2.53 ± 0.05 mm/day), *Hymenochaete tropica* LE-BIN 5047 (0.94 ± 0.27 mm/day) and *Hymenochaete rheicolor* LE-BIN 5115 (2.44 ± 0.24 mm/day), *Ganoderma flexipes* LE-BIN 5120 (0.82 ± 0.08 mm/day), or *Favolus tenuiculus* LE-BIN 5130 (2.32 ± 0.24 mm/day) and some others. Most of the studied marsupial fungi grew slowly in culture. More data on the growth rate of the studied strains are given in Table S2.

Macromycetes are the most valuable producers of oxidases such as polyphenol oxidase, laccase, peroxidase, tyrosinase and other oxidizing enzymes. These enzymes have wide applications in many areas of human life and industry, including pulping, the decolorization and detoxification of textile waste, wastewater treatment, and the bioremediation of contaminated soils [48,49,50,51,52]. Strains from the LE-BIN collection have been consistently used to screen and study oxidoreductases for many years, and more than 550 strains from approximately 350 species of aphyllophoroid and agaricoid fungi in the collection have been involved in oxidase studies [16]. However, tropical strains were not used in that study. Despite the growing interest in studying fungi from tropical regions, the ability of these fungi to produce lignocellulolytic enzymes is not well understood [1,2,53].

**Figure 5:**
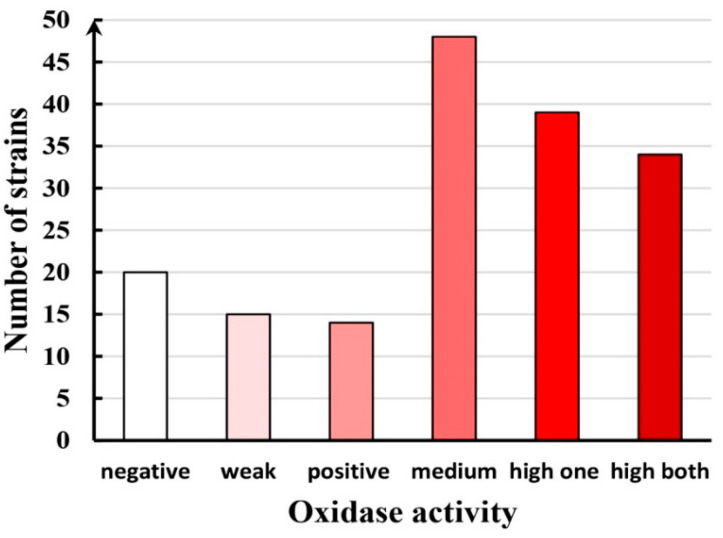
Oxidative potential of macromycetes from Vietnam: number of strains tested with negative (−), weak (+/−), positive (+), medium (++), high in one of the substrates (+++/++), high in both substrates (+++).

In total, 170 macromycetes strains from Vietnam were studied for oxidase activity by the express method using syringaldazine (SG) to characterize laccase and peroxidase activities and guiacol (GU) to characterize nonspecific oxidases. The majority of studied strains were xylotrophic fungi. The results of the screening showed that 135 strains (79%) of studied basidiomycete strains revealed positive activity oxidizing at least one of used substrates. High activity (+++) on both substrates was observed in 34 strains (20%) and 39 strains (23%) showed high activity on at least one substrate. Negative (−) or weak (+/−) activity on both substrates revealed for 35 strains (21%). The oxidative potential of macromycete strains from Vietnam is demonstrated in Figure 5. The results obtained for the strains studied are presented in the Supplementary Materials (Table S2).

Among the fast-growing species with high oxidase activity on both substrates were the aphyllophoroid fungi *Theleporus membranaceus* LE-BIN 5170 (8.16 ± 0.07 mm/day), *Cellulariella acuta* LE-BIN 4545 (7.17 ± 0.56 mm/day), *Irpicodon* sp. LE-BIN 4303 (7.17 ± 0.45 mm/day), and *Duportella tristicula* LE-BIN 5035 (6.27 ± 1.27 mm/day). Agaricoid fungus *Pleurotus pulmonarius*, strain LE-BIN 4591 with a growth rate of 6.14 ± 0.12 mm/day also showed high oxidase activity. A number of fast-growing strains, i.e., *Cerrena zonata* LE-BIN 4492, *Trametes versicolor* LE-BIN 4516 and LE-BIN 4557, *Phlebia acerina* LE-BIN 4584, *Fulvifomes fastuosus* LE-BIN 4531, *Lentinus crinitus* LE-BIN 4255, *Funalia floccosa* LE-BIN 4534 showed high activity (+++) on one of the substrates while on the other substrate they showed medium (++) activity. It should be noted that strains were found that showed high activity for one substrate but either no activity (−) or very low activity (+/−) for the other substrate: *Neofomitella fumosipora* LE-BIN 4575—high activity on syringaldazine (+++) and negative (−) on guiacol; *Grammothele lineata* LE-BIN 4219 and *Coriolopsis brunneoleuca* LE-BIN 5074—high activity on syringaldazine (+++) and low (+/−) on guiacol. It was also found that a number of slow-growing strains of both aphyllophoroid and agaricoid fungal species had high oxidative potential, e.g., *Hypholoma fasciculare* LE-BIN 4583 (1.30 ± 0.02 mm/day), *Gymnopus* sp. LE-BIN 5118 (1.28 ± 0.04 mm/day), *Mycena jingyinga* LE-BIN 4556 (1.26 ± 0.04 mm/day), *Ganoderma flexipes* LE-BIN 5120 (0.82 ± 0.08 mm/day), *Cyptotrama asprata* LE-BIN 5145 (0.5 ± 0.04 mm/day), *Gymnopus* sp. LE-BIN 5036 (0.48 ± 0.02 mm/day) and some others.

**Figure 6:**
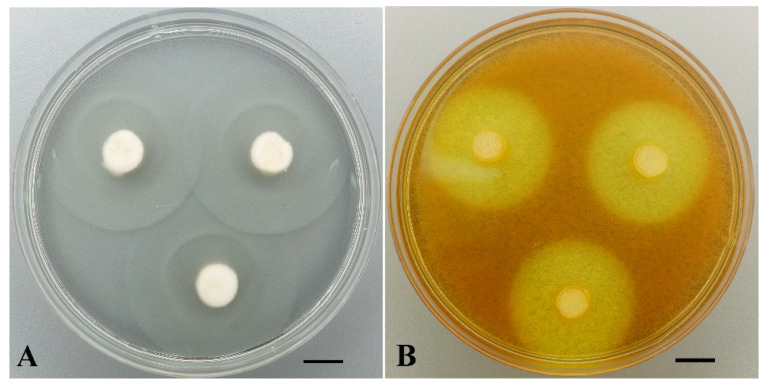
(**A**) Lysis zones on gelanine substrate by *Candolleomyces* aff. *candolleanus* LE-BIN 5112 revealing its high proteolytic potential. (**B**) Zones on MCC substrate revealing high cellulolytic potential of *Phlebia acerina* LE-BIN 4498. Scale bars are 10 mm.

The proteolytic potential of 67 tropical strains of different taxa was evaluated using gelatin substrate. Positive activity was observed in 59 strains, and only 8 strains showed an almost lack of activity—very weak lysis of the substrate was only under inoculated disks, 32 strains formed lysis zones from 13.7 ± 0,5 to 25 ± 0.1 mm in diameter, and 27 strains showed high activity—lysis zones exceeded 25 mm. The most active strains were *Truncospora tephropora* LE-BIN 5184, *Hymenochaete* sp. LE-BIN 5172, *Hypholoma* sp. LE-BIN 5179 and *Cruentomycena* sp. LE-BIN 5157, whose activity resulted in complete lysis of the substrate in Petri dishes. It should be noted that the last three strains proved to be slow-growing cultures. The results on gelatin lysis obtained for the strains studied are presented in the Supplementary Materials (Table S2). An example of high GA demonstrated by *Candolleomyces* aff. *candolleanus* LE-BIN 5112 is shown in Figure 6A.

**Figure 7:**
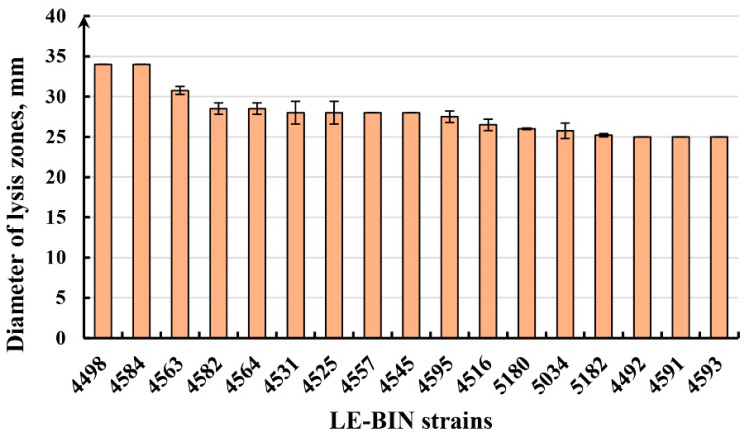
Cellulolytic activity of selected fast growing strains: *Phlebia acerina* LE-BIN 4498 and LE-BIN 4584, *Xylobolus spectabilis* LE-BIN 4563, *Ganoderma applanatum* LE-BIN 4582, *Psathyrella* sp. LE-BIN 4564, *Fulvifomes fastuosus* LE-BIN 4531, *Trametes maxima* LE-BIN 4525, *Trametes versicolor* LE-BIN 4557, *Cellulariella acuta* LE-BIN 4545, *Trametes sanguinea* LE-BIN 4595, *Trametes versicolor* LE-BIN 4516, *Tinctoporellus epimiltinus* LE-BIN 5180, *Flavodon flavus* LE-BIN 5034, *Trametes meyenii* LE-BIN 5182, *Cerrena zonata* LE-BIN 4492, *Pleurotus pulmonarius* LE-BIN 4591, *Conchomyces* sp. LE-BIN 4593.

A total of 131 macromycete strains from different taxa were examined for cellulolytic activity (CA) on MCC substrate. Of these, 115 strains showed activity with zones more than 15 mm in diameter, six strains showed low activity with zones 10–15 mm in diameter, and nine strains showed only traces. High activity with zones of 25 mm or more in diameter was shown by 43 strains. More than 30 mm was observed in six strains: *Phlebia acerina* LE-BIN 4498 (34 ± 0 mm) and LE-BIN 4584 (34 ± 0 mm), *Microporus xanthopus* LE-BIN 4517 (32 ± 0 mm), *Vararia* sp. LE-BIN 4559 (31.5 ± 0.71 mm), *Xylobolus spectabilis* LE-BIN 4563 (30.75 ± 0.5 mm) and *Trametes hirsuta* LE-BIN 5064 (30.66 ± 0.41 mm). In addition, *Phlebia acerina* and *Xylobolus spectabilis* strains were characterized by rapid growth (8.63 ± 0.30, 8.6 ± 0.17 and 7.88 ± 1.01 mm/day, respectively). An example of cellulolytic activity on MCC substrate is presented in Figure 6B. The diagram (Figure 7) shows fast growing strains with high cellulolytic activity. More data on cellulolytic activity of the tropical strains are presented in the Supplementary Materials (Table S2).

Although the search for basidial fungi with high oxidative and cellulolytic activity has been the subject of many studies [16,53,54,55,56,57,58,59], analyzing the enzymatic activity of tropical species remains a challenging task. This study presents data on a large number of previously unstudied species from the tropical region. Based on the results obtained, the strains with high enzymatic activity are summarized in Table 5. in order to present promising macromycete species with high biosynthetic potential. To the best of our knowledge, *Truncospora tephropora*, *Coriolopsis brunneoleuca*, *Cerrena zonata*, *Fulvifomes fastuosus*, and *Xylobolus spectabilis* have not been previously studied for enzymatic activities or other medically and biotechnologically relevant properties.

Most of the strains with high activity belong to aphyllophoroid fungi, mainly white rot fungi. Only *Candolleomyces* aff. *candolleanus* and *Lentinus squarrosulus* were selected from the group of agaricoids. Among the marsupial macromycetes, *Xylaria grammica* and *Nemania* sp. appear to be promising for further study. The obtained data confirmed the high biotechnological potential of the *Trametes* species (*T. versicolor*, *T. sanguinea* and *T. meyenii*) and drew attention to the biotechnological potential of *Phlebia acerina*, *Fulvifomes fastuosus*, *Cellulariella acuta*, *Truncospora tephropora*, *Tinctoporellus epimiltinus*, *Theleporus membranaceus*, *Cerrena zonata* and other fast growing less studied species.

Analysis of the resource potential of macromycetes from national parks in Vietnam confirms the importance of ex situ conservation work on the fungal diversity of tropical regions. Macromycetes in culture collections are a valuable source for scientific and commercial applications [4]. A study of tropical strains preserved in the Komarov Botanical Institute Basidiomycetes Culture Collection showed that 53 of the 180 strains studied showed high growth rates, which makes them useful for cultivation for biotechnological purposes as a source of various bioactive compounds. High cellulolytic activity was exhibited by 43 strains. This aspect makes them promising candidates for application in bioconversion processes, which is particularly important in the light of global challenges of biomass utilization and sustainable resource management [52,53,54]. In addition, 73 strains showed high activity of oxidizing enzymes. This opens up possibilities for testing them in various biotechnological processes such as bioremediation, decolorization and bioproducts industry [4,49,50,51]. It is also worth noting that 27 strains showed high proteolytic activity (lysis zones over 25 mm in diameter). Proteins are the main source of organic nitrogen in forest soils. This nitrogen is made available to trees through the depolymerizing activity of symbiotic ectomycorrhizal fungi. However, the mechanisms by which these fungi depolymerize proteins and assimilate the released nitrogen remain poorly understood. The identified producers of extracellular proteolytic enzymes can be good models to study the degradation pathways of extracellular proteins in more detail. Proteolytic enzymes of macrofungi can also be studied for the production of enzymes for food and feed industries, as well as in other areas where degradation of protein compounds is required [4].

As producers of proteins, polysaccharides (such as beta-glucans) [60,61,62,63] and numerous secondary metabolites such as terpenoids, sterols, flavonoids, alkaloids, lectins, steroids, phenols, triterpenoids, etc. [64,65], many basidiomycete species possess a wide range of pharmacological activities, including antioxidant, antiallergic, antibacterial, antiviral, anti-inflammatory, immunomodulatory, antitumor, hepatoprotective, hypolipidaemic, hypotensive, antidiabetic, neuroprotective, nephroprotective, and osteoprotective activities, and are natural sources for the pharmaceutical industry [64,66,67]. However, the medicinal properties of fungi have only been studied in detail on a limited number of species, despite the fact that more than 700 species of fungi with different biological activities have already been discovered [68]. Moreover, Hyde et al. [10] noted that during their 10 years of research in Northern Thailand, in addition to numerous taxonomic novelties, they obtained many new and unique bioactive secondary metabolites from various fungi. In addition, the producer organisms often turned out to be as yet undescribed species. Thus, there is reason to believe that the collection of pure fungal cultures from Vietnam could also serve as a source of new chemical compounds for years ahead. Therefore, the collection of tropical strains that includes previously unstudied species is a useful resource for the investigation of new medicinal properties and bioactive substances. The study of these tropical strains may lead to the discovery of unique compounds with potential medicinal properties such as anti-inflammatory, antioxidant, anticancer, and many other activities.

The results of our study highlight the diversity of cultivated fungi in Vietnam and the enzymatic potential inherent in tropical basidial fungi. This diversity opens new horizons for further research aimed both at fundamental studies of the physiological mechanisms of fungi and at optimizing cultivation conditions and increasing the yield of target products.

## 4. Conclusions

A large diversity of macromycetes from eight protected areas of Vietnam was collected and preserved ex situ, verified by barcoding, studied for its bioresource potential and presented to the scientific community for the first time.

Specimens of species potentially new to science were collected and studied. Their ITS sequences were deposited and released for public in the NCBI GenBank. Such specimens and cultures are of particular interest for taxonomic studies.

Tropical strains of macrofungi maintained in the LE-BIN Culture Collection represent a valuable resource for biotechnology and medicine, and their cellulolytic, oxidative, and proteolytic activities offer new opportunities for sustainable biomass management, obtaining new enzymes and developing efficient technologies for plant waste processing.

## Figures and Tables

**Table 1:** Origination of tropical strains from Vietnam.

Vietnam Protected Territories	Collection Dates
NP Phu Quoc	18–24 October 2012
NP Cat Tien	28–31 October 2012; 5–12 May 2014; 6–20 February 2021; 20–21 November 2022
NP Bidoup—Nui Ba	2–8 November 2012; 23–29 May 2014; 29 October–6 November 2019; 3–11 July 2023; 19–30 October 2023
NP Bu Gia Map	13–18 May 2014; 25 October–2 November 2018; 21–24 October 2019
NR Kon Chu Rang	25 February–3 March 2021; 23–28 October 2022; 14–20 July 2023; 4–6 November 2023
NP Chu Yang Sin	16–27 May 2019
NP Ta Dung	3–19 October 2022
NP Phia Oac—Phia Den	7–17 November 2018; 4–13 June 2019; 4–12 October 2019

**Table 2:** Ex situ conservation of macromycetes in Vietnam.

Vietnam Protected Territories	Number of Specimens		Number of LE-BIN Strains
	Collected	Isolates	
NP Phu Quoc	88	50	41
NP Cat Tien	189	127	92
NP Bidoup—Nui Ba	445	279	184
NP Bu Gia Map	224	137	127
NR Kon Chu Rang	191	103	80
NP ChuYang Sin	58	33	14
NP Ta Dung	165	115	104
NP Phia Oac—Phia Den	247	160	143
Total:	1519	1004	785

**Table 3:** Systematic arrangement of the LE-BIN strains from Vietnam. The number of species and strains (in brackets) is provided after the genus name.

Class	Order	Family	Genus
**ASCOMYCOTA**			
Leotiomycetes	Helotiales	**Chlorociboriaceae**	Chlorociboria 1 (1)
		**Chlorospleniaceae**	Chlorosplenium 1 (1)
		**Gelatinodiscaceae**	Ascocoryne 1 (1)
		**Helotiaceae**	Dicephalospora ≥ 1 (3), Hymenoscyphus 1 (1), Tatraea ≥ 1 (3)
Orbiliomycetes	Orbiliales	**Orbiliaceae**	Orbilia 1 (1)
Pezizomycetes	Pezizales	**Sarcoscyphaceae**	Cookeina 2 (2)
		**Sarcosomataceae**	Plectania ≥ 1 (2)
Sordariomycetes	Hypocreales	**Cordycipitaceae**	Cordyceps 1 (1), Isaria 1 (1)
	Xylariales	**Xylariaceae**	Durotheca 1 (1), Nemania 1 (1), Xylaria ≥ 6 (14)
**BASIDIOMYCOTA**			
Agaricomycetes	Agaricales	**Agaricaceae**	Agaricus ≥ 1 (5), Leucoagaricus 1 (1), Leucocoprinus 1 (1), Micropsalliota ≥ 1 (2)
		**Bolbitiaceae**	Conocybe 1 (1)
		**Callistosporiaceae**	Callistosporium 1 (1)
		**Campanellaceae**	Campanella ≥ 1 (5), Tetrapyrgos ≥ 1 (4)
		**Clitocybaceae**	Clitocybe 1 (1), Collybia 1 (1)
		**Crepidotaceae**	Crepidotus 1 (1)
		**Cystostereaceae**	Crustomyces 1 (1)
		**Entolomataceae**	Clitopilus 2 (2)
		**Fayodiaceae**	Conchomyces ≥ 1 (2)
		**Hymenogastraceae**	Gymnopilus ≥ 2 (7), Psilocybe ≥ 1 (4)
		**Lyophyllaceae**	Tephrocybe 1 (1), Termitomyces (1)
		**Marasmiaceae**	Chaetocalathus 1 (1), Crinipellis ≥ 1 (3), Marasmius ≥ 2 (25), Paramarasmius 2 (2)
		**Mycenaceae**	Cruentomycena 1 (1), Cynema 1 (3), Favolaschia ≥ 4 (16), Filoboletus ≥ 1 (2), Mycena ≥ 4 (25), Panellus ≥ 3 (19), Resinomycena 1 (1)
		**Nidulariaceae**	Cyathus 1 (1), Nidula 1 (1), Nidularia 1 (1)
		**Omphalotaceae**	Anthracophyllum ≥ 1 (3), Collybiopsis ≥ 3 (24), Gymnopus ≥ 2 (10), Lentinula 3 (5), “Marasmiellus” 2 (4), Mycetinis ≥ 1 (8), Neonothopanus 1 (10), Omphalotus ≥ 1 (2), Pseudomarasmius 1 (2)
		**Phyllotopsidaceae**	Phyllotopsis 1 (1)
		**Physalacriaceae**	Cyptotrama ≥ 2 (6), Flammulina 1 (1), Gloeocephala 1 (1), Hymenopellis ≥ 1 (4), Mucidula ≥ 1 (7), Oudemansiella ≥ 1 (2), Xerula ≥ 1 (7)
		**Pleurotaceae**	Hohenbuehelia ≥ 1 (2), Pleurotus ≥ 4 (12)
		**Porotheleaceae**	Clitocybula ≥ 1 (2), Gerronema ≥ 1 (3), Hydropus ≥ 1 (8), Pseudohydropus 1 (1), Trogia ≥ 1 (4), Vanromburghia 1 (1)
		**Psathyrellaceae**	Candolleomyces ≥ 1 (5), Coprinellus ≥ 1 (2), Coprinopsis ≥ 1 (3), Psathyrella ≥ 1 (4)
		**Pterulaceae**	Pterulicium 1 (1),
		**Radulomycetaceae**	Radulomyces ≥ 1 (2)
		**Resupinataceae**	Resupinatus 1 (1)
		**Schizophyllaceae**	Schizophyllum 1 (1)
		**Strophariaceae**	Deconica 1 (1), Hypholoma ≥ 2 (12), Kuehneromyces 1 (1), Melanotus ≥ 1 (4), Pholiota ≥ 1 (5)
		**Xeromphalinaceae**	Heimiomyces 1 (1), Xeromphalina ≥ 1 (4)
	Amylocorticiales	**Amylocorticiaceae**	Irpicodon 1 (1)
	Atheliales	**Atheliaceae**	Athelia 1 (1)
	Auriculariales	**Auriculariaceae**	Auricularia ≥ 1 (13), Eichleriella 1 (1), Elmerina 2 (2), Protodaedalea 1 (1)
	Cantharellales	**Hydnaceae**	Rogersiomyces 1 (1)
	Gloeophyllales	**Gloeophyllaceae**	Hispidaedalea 1 (1)
	Hymenochaetales	**Hirschioporaceae**	Pallidohirschioporus ≥ 1 (3)
		**Hymenochaetaceae**	Erythromyces 1 (1), Fulvifomes ≥ 1 (2), Fuscoporia ≥ 2 (4), Hymenochaete ≥ 5 (8), Phellinus ≥ 1 (6), Pyrrhoderma 2 (2)
		**Hyphodontiaceae**	Hyphodontia 1 (1)
		**Rigidoporaceae**	Rigidoporus ≥ 1 (2)
		**unknown**	Trichaptum s. l. (4)
	Phallales	**Phallaceae**	Phallus 1 (1)
	Polyporales	**Cerrenaceae**	Cerrena ≥ 1 (5)
		**Fomitopsidaceae**	Antrodia 1 (1), Fomitopsis ≥ 1 (4), Rhodofomitopsis 1 (2)
		**Ganodermataceae**	Ganoderma ≥ 6 (23), Sanguinoderma 1(1)
		**Hypochniciaceae**	Hypochnicium 1 (1)
		**Incrustoporiaceae**	Incrustoporia 1 (1), Skeletocutis 1 (1)
		**Irpicaceae**	Efibula 1 (1), Flavodon 1 (5), Gloeoporus 1 (1), Irpex 1 (1)
		**Laetiporaceae**	Laetiporus 1 (1)
		**Meripilaceae**	Physisporinus ≥ 2 (6)
		**Meruliaceae**	Climacodon 1 (1), Crustodontia (1), Merulius 1 (1), Mycoacia ≥ 1 (2), Mycoaciella 1 (1), Phlebia 1 (3), Scopuloides 1 (1)
		**Panaceae**	Cymatoderma 2 (4), Panus ≥ 2 (8)
		**Phaeolaceae**	Phaeolus 1 (1)
		**Phanerochaetaceae**	Bjerkandera 1 (3), Oxychaete 1 (1), Phlebiopsis ≥ 1 (2), Terana 1(1)
		**Polyporaceae**	Abundisporus 1 (1), Cellulariella 1 (5), Coriolopsis 1 (2), Cubamyces 3 (4), Earliella 1 (2), Favolus ≥ 4 (9), Fomes 1 (1), Fomitella 1 (1), Funalia 2 (2), Grammothele 1 (2), Hexagonia ≥ 1 (3), Lentinus ≥ 6 (19), Lopharia ≥ 1 (2), Megasporoporiella 1 (1), Microporellus 1 (1), Microporus ≥ 2 (25), Neofomitella 2 (3), Perenniporiopsis 1 (1), Picipes ≥ 1 (3), Podofomes 1 (1), Polyporus ≥ 1 (5), Porogramme 1 (1), Theleporus 1 (1), Tinctoporellus 1 (2), Trametes ≥ 11 (36), Truncospora 1 (2)
		**Steccherinaceae**	Junghuhnia 1 (1), Nigroporus 1 (7), Steccherinum ≥ 3 (6)
	Russulales	**Auriscalpiaceae**	Artomyces ≥ 1 (2), Auriscalpium 1 (1)
		**Bondarzewiaceae**	Bondarzewia 1 (1)
		**Hericiaceae**	Dentipellis ≥ 1 (2)
		**Peniophoraceae**	Duportella 1 (1), Vararia ≥ 1 (2)
		**Stereaceae**	Stereum ≥ 3 (23), Xylobolus ≥ 3 (10), Megalocystidium 1 (1)
		**Wrightoporiaceae**	Wrightoporia 1 (1)
Dacrymycetes	Dacrymycetales	**Dacrymycetaceae**	Dacrymyces 1 (2)
Tremellomycetes	Tremellales	**Phaeotremellaceae**	Phaeotremella 1 (1)

**Table 4:** Species represented by the greatest number of strains.

Species	Strain Number
*Neonothopanus nambi*	10
*Microporus xanthopus*	9
*Favolaschia manipularis*	8
*Trametes vernicipes*	8
*Nigroporus vinosus*	7
*Stereum ostrea*	7
*Auricularia delicata*	6
*Microporus affinis*	6
*Pleurotus pulmonarius* s. l.	6
*Trametes versicolor*	*6*
*Candolleomyces candolleanus* s. l.	5
*Cellulariella acuta*	5
*Flavodon flavus*	5
*Lentinus arcularius* s. l.	5
*Mucidula mucida*	5
*Panellus luxfilamentus*	5
*Xylobolus princeps*	5

**Table 5:** Growth rate and enzymatic activity of selected LE-BIN strains from Vietnam.

Taxon, Strain Num.	Growth Rate (mm/d)	Oxidases (SG)	Oxidases (GU)	Proteinases (GA, mm)	Cellulases (CA, mm)
*Phlebia acerina* LE-BIN 4498	8.63 ± 0.30	++ *	++	—	34 ± 0
*Phlebia acerina* LE-BIN 4584	8.6 ± 0.17	+++	++	—	34 ± 0
*Xylobolus spectabilis* LE-BIN 4563	7.88 ± 1.01	+	+++	—	30.75 ± 0.5
*Fomitopsis* sp. LE-BIN 5106	4.33 ± 0.57	−	−	31.9 ± 0.7	29.3 ± 0.4
*Fulvifomes fastuosus* LE-BIN 4531	7.21 ± 0.16	++	+++	—	28.00 ± 1.41
*Trametes versicolor* LE-BIN 4557	6.18 ± 0.38	+++	++	—	28 ± 0
*Cellulariella acuta* LE-BIN 4545	7.17 ± 0.56	+++	+++	—	28 ± 0
*Trametes sanguinea* LE-BIN 4595	6.14 ± 0.09	+++	+	—	27.5 ± 0.71
*Trametes versicolor* LE-BIN 4516	8.6 ± 0.21	+++	++	—	26.5 ± 0.71
*Trametes meyenii* LE-BIN 5182	10.55 ± 0.42	—	—	25 ± 0.1	25.2 ± 0.2
*Xylaria grammica* LE-BIN 5102	2.42 ± 0.26	+	+++	45 ± 0.7	24.5 ± 0.3
*Truncospora tephropora* LE-BIN 5184	6.24 ± 0.05	—	—	Full lysis	23.4 ± 0.3
*Candolleomyces* aff. *candolleanus* LE-BIN 5112	6.77 ± 0.08	++	−	44.2 ± 0.6	23 ± 0.5
*Lentinus squarrosulus* LE-BIN 5189	7.69 ± 0.09	−	+/−	27.2 ± 0.2	20.5 ± 0.1
*Nemania* sp. LE-BIN 5155	4.64 ± 0.04	+/−	−	26 ± 1.1	18.7 ± 0.2
*Auricularia delicata* LE-BIN 5164	3.25 ± 0.07	++	++	25.4 ± 1.4	24.7 ± 0.4
*Tinctoporellus epimiltinus* LE-BIN 5180	10.82 ± 0.12	—	—	23.7 ± 0.3	26 ± 0.1
*Theleporus membranaceus* LE-BIN 5170	8.16 ± 0.07	+++	+++	18.4 ± 0.6	22.9 ± 0.6
*Coriolopsis brunneoleuca* LE-BIN 5131	3.97 ± 0.55	++	+++	16.6 ± 0.9	27.8 ± 0.6
*Cerrena zonata* LE-BIN 4492	8.6 ± 0.34	++	+++	—	25 ± 0

* Note: Oxidase activity was expressed as follows “–“—negative, “+/–“—weak, “+”—positive, “++”—medium, “+++”—high.

## Data Availability

Data are contained within the article and Supplementary Materials (Tables S1 and S2).

## Supplementary Materials

The following supporting information can be downloaded at https://www.mdpi.com/article/10.3390/jof11010034/s1. The strains deposited to the GenBank with the NCBI accession numbers are presented in the Supplementary Materials (Tables S1 and S2).
